# Optimization of Extraction Parameters by Using Response Surface Methodology, Purification, and Identification of Anthocyanin Pigments in *Melastoma malabathricum* Fruit

**DOI:** 10.1155/2013/810547

**Published:** 2013-09-23

**Authors:** Nordiyanah Anuar, Ahmad Faris Mohd Adnan, Naziz Saat, Norkasmani Aziz, Rosna Mat Taha

**Affiliations:** ^1^Institute of Biological Sciences, Faculty of Science, University of Malaya, 50603 Kuala Lumpur, Malaysia; ^2^Centre for Ionics, Department of Physics, University of Malaya, 50603 Kuala Lumpur, Malaysia

## Abstract

Anthocyanins not just have various benefits in food industry but also have been used as natural colourants in cosmetic, coating products and as potential natural photosensitizers in solar cell. Thus, the main purpose of this study was to obtain information on the maximum yield of anthocyanin that can be recovered from *Melastoma malabathricum* fruit. Factors such as extraction temperature, extraction time, and solid to liquid ratio were identified to be significantly affecting anthocyanin extraction efficiency. By using three-level three-factor Box-Behnken design, the optimized conditions for anthocyanin extraction by acidified methanol (*R* ^2^ = 0.972) were temperature of 60°C, time of 86.82 min, and 0.5 : 35 (g/mL) solid to liquid ratio while the optimum extraction conditions by acidified ethanol (*R* ^2^ = 0.954) were temperature of 60°C, time of 120 min, and 0.5 : 23.06 (g/mL) solid to liquid ratio. The crude anthocyanin extract was further purified by using Amberlite XAD-7 and Sephadex LH-20 column chromatography. Identification of anthocyanins revealed the presence of cyanidin dihexoside, cyanidin hexoside, and delphinidin hexoside as the main anthocyanins in *M. malabathricum* fruit.

## 1. Introduction

Anthocyanins are identified as water soluble compounds having molecular structure based on a C_6_-C_3_-C_6_ skeleton. Anthocyanins are the most conspicuous subset under flavonoid group, due to the wide range of colours resulting from their synthesis [1]. They are responsible for the red, purple, and blue colours in many parts of the plants. Today, the interest in anthocyanins has arised due to their unique structure and beneficial health claims food and pharmaceutical industry have greatly benefited from. They have been used for various food preparations such as jelly dessert, milk dessert, soft ice-cream, hard ice-cream, and yogurt [2]. Besides being important in food and pharmaceutical industry, various natural dye extracts which include anthocyanins have also been actively explored by researchers to be used as sensitizer in dye-sensitized solar cells [3, 4] and coating [5].

These valuable bioactive compounds also have been reported to have biological effects on the physiological functions of cells such as reducing oxidative cell damage and increasing high density lipoprotein (HDL) cholesterol level [6, 7]. Anthocyanins with an ortho-dihydroxyphenyl structure on the B-ring (e.g., delphinidin) may contribute to the induction of apoptosis on which its chemo preventive action against cancer is based [8]. Recently, researchers have found that cyanidin-3-glucoside could improve functional recovery of neurological dysfunction in a rat model having traumatic spinal cord injury while delphinidin-3-glucoside was found to have significant roles against thrombosis and cardiovascular diseases [9, 10].

*Melastoma malabathricum*, which belongs to the family Melastomataceae, is described as a flowering shrub that is distributed widely in South and Southeast Asia [11]. The fruits are considered as a rich source of anthocyanins as the fruits turned to dark purple when ripe. Previous works done on *M. malabathricum *were primarily on thephytochemical contents and medicinal properties obtained from the leaves, stems, and roots [12–14]. This shrub has the potential as a steady supply of feedstock for pigment production.

Extraction of the bioactive compound is influenced by various process parameters such as solvent composition, pH, temperature, extraction time, and solid to liquid ratio [15, 16]. Extracts rich in anthocyanins were usually extracted using methanol, ethanol, acetone, water, or mixtures and with the addition of small amount of acid which is recommended to prevent the degradation of the nonacylated compounds. Response surface methodology (RSM) is an economically efficient experimental procedure for optimizing this complex process. Compared to using “one to one factor” method, RSM is far better since in RSM the several process variables simultaneously interact with each other. This method is based on multivariate nonlinear model that has been widely used in chemical, biological, and agricultural applications to predict the optimal conditions of the systems. 

To the best of our knowledge, there were no studies focusing on optimizing the parameters for anthocyanin extraction from *M. malabathricum* fruit. Thus, the objectives of this study were to obtain maximum yield of anthocyanin recovery from *M. malabathricum* fruit and also to identify the major anthocyanin components in *M. malabathricum* fruit using UPLC-ESI-MS/MS.

## 2. Materials and Methods

### 2.1. Materials

The chemical reagents (potassium chloride, sodium acetate) used in this study were of analytical grade and obtained from Sigma Aldrich. Methanol (99.8%) and undenatured ethanol (99.8%) from Systerm were used as extraction solvents. Ethyl acetate (Systerm) was used in the separation process. Formic acid, ammonium formate, and acetonitrile were of HPLC grade obtained from Merck. Sephadex LH-20 and Amberlite XAD-7 from GE Healthcare were used as chromatography resin. Deionized water used in this study was purified at 18.2 MΩ.cm^−1^ (Barnstead RO & Deionized Systems).

### 2.2. Methods

#### 2.2.1. Sample Preparation

Fresh mature fruits of *M. malabathricum* were used as raw material and were peeled prior to freeze-drying. The freeze-dried fruits were turned into powder by using a commercial grinder. The powder was sieved using sieve number 60 (250 *μ*m) to achieve constant particle size. All samples were kept at −20°C in amber bottle and kept until further analysis.

#### 2.2.2. Extraction of Pigments

In the extraction procedure, 0.5 g of the fruit powder was mixed with various volumes of methanol acidified with 0.5% acetic acid or ethanol acidified with 0.5% acetic acid to give a solid to liquid ratio ranging from 0.5 : 5 to 0.5 : 35 (g/mL). Conical flask was used and covered with aluminium foil to prevent the evaporation of solvent. The flask containing sample powder along with solvent was incubated in thermostatic water bath at various temperatures (30–60°C) and various time intervals (60–120 min). After extraction for a period of selected time, the mixture was centrifuged for 10 min. The supernatant was then filtered and evaluated for the total anthocyanin content. Experiments were performed in randomized order to minimize the variability caused by nuisance factors. All the experiments were performed in triplicate and the average value was used for the determination of total anthocyanin content from *M. malabathricum* fruit. 

#### 2.2.3. Experimental Design

RSM was used to optimize the methanolic extraction and ethanolic extraction of anthocyanins from *M. malabathricum* fruit. A Box-Behnken design (BBD) was used in the optimization of process variables with three factors at three levels with 17 runs, including 5 central points (Table 1). The responses function (*Y*) was partitioned into linear, quadratic, and interactive components. Experimental data were fitted to the second-order regression equation:

(1) Y=b0+b1X1+b2X2+b3X3+b11X12 +b22X22+b33X32+b12X1X2+b13X1X3 +b23X2X3,

where *b*_0_ is the intercept; *b*_1_, *b*_2_, and *b*_3_ are linear coefficients; *b*_11_, *b*_22_, and *b*_33_ are squared coefficients; *b*_12_, *b*_13_, and *b*_23_ are interaction coefficients. 

The experimental design and statistical analysis were performed using Design-Expert software (version 8.0.7.1, Stat Ease Inc., Minneapolis, MN, USA). The model adequacies were checked in terms of the values of *R* ^2^ and adjusted *R* ^2^. Analysis of variance (ANOVA) was employed to determine the significance of the models. Verification of optimized conditions and predicted values were done in triplicate to confirm the validity of the models.

#### 2.2.4. Total Anthocyanin Content Measurement

The total anthocyanin content was determined according to the spectrophotometric pH differential method [17]. Samples were diluted separately with 0.025 M potassium chloride buffer (pH 1) and 0.4 M sodium acetate buffer (pH 4.5). Absorbance of the mixture was measured at 511 (*λ*_vis-max_) and 700 nm using a UV-Vis spectrophotometer. Absorbance was calculated as *A* = [(*A*_511_ − *A*_700_) pH 1.0 − (*A*_511_ − *A*_700_) pH 4.5]. The total anthocyanin content was calculated as cyanidin-3-glucoside equivalents as in the following equation:

(2) Anthocyanin  content  (mg/100 g)  =A×MW×DF×V×100ε×l×msample,

where *A* is the absorbance, MW is the molecular weight of (449.2 g/mol of cyanidin-3-glucoside), DF is the dilution factor, *V* is the solvent volume (mL) that was brought as sample stock solution, *ε* is the molar absorptivity (26900), *l* is the cell path length (1 cm), and *m* is the freeze-dried sample weight (g).

#### 2.2.5. Purification of Anthocyanin

The crude anthocyanin extract was concentrated by using a rotary evaporator (40°C). The aqueous concentrates were then placed in a separating funnel and an equal volume of ethyl acetate was added to remove lipids, chlorophylls, and other nonpolar compounds from the mixture. The partitioned aqueous extract was further purified by using ion exchange chromatography (IEC) and size exclusion chromatography (SEC) using Amberlite XAD-7 resin and Sephadex LH-20 as separation matrixces, respectively. Anthocyanin content in the fractions collected using size exclusion chromatography was determined by pH differential method and fractions containing the highest content of anthocyanin were chosen for identification analyses.

#### 2.2.6. Identification of Anthocyanin Using UPLC-ESI-MS/MS

Analytical ultra performance liquid chromatography (Perkin Elmer FX15) was used in this study. The anthocyanin fractions were then analyzed using AB Sciex 3200Q Trap, equipped with Phenomenex Aqua C18 reverse-phase column (50 mm × 2.0 mm × 5 *μ*M). Solvents were (A) water with 0.1% formic acid and 5 mM ammonium formate and (B) acetonitrile with 0.1% formic acid and 5 mM ammonium formate, establishing the following gradient: from 10% B to 90% B from 0.01 min to 8 min, held for 3 min and back to 10% A in 0.1 min and reequilibrated for 5 min. Samples were filtered with nylon 0.22 *μ*M. The electrospray ionization (ESI) was operated in negative and positive ion modes under the following conditions: mass range between 100 and 1200; capillary voltages +5500 V and −4500 V; nebulizer purified N_2_ gas, 40 psi, and source temperature 400°C. Mass fragmentations were based on journal references and ACD/Labs advanced chemometrics mass fragmentation predictive software [6, 18].

## 3. Results and Discussion

### 3.1. Box-Behnken Analysis

In this study, BBD was used for response surface optimization with three process variables (extraction temperature, extraction time, and solid to liquid ratio) at three levels. Designs using BBD are usually very efficient in terms of the number of required runs and therefore are less expensive to run compared to central composite design (CCD). The design points fall within a safe operating limit, within the nominal high and low levels, as BBD does not contain any points at the vertices of the cubic region. This could be advantageous when the factor-level combinations are prohibitively expensive or impossible to test because of the physical process constraints [19]. 

Two different tests, namely, sequential model sum of squares and model summary statistic were performed to check the adequacy of the models generated from the obtained data and the results are given in Table 2. Model summary statistics output (Table 2) showed that, for methanolic extraction and ethanolic extraction, the values for the *R* ^2^ and adjusted *R* ^2^ were the highest compared the other models while the cubic model was disregarded as it is aliased. For quadratic versus 2FI (2 factor interaction), the *P* value obtained was less than 0.0001 which shows strength of significance. The addition of the quadratic (squared) term to the mean, linear, and the two-factor interaction terms would only strengthen the model. With the exclusion of the cubic model, the Box-Behnken matrix has sufficient data to interpret the outcome of the present system [20]. 

### 3.2. Statistical Analysis for Selected Models

Summary of analysis of variance (ANOVA) for the selected quadratic polynomial model for methanolic extraction and ethanolic extraction was listed in Table 3. The ANOVA of quadratic regression model demonstrated that both models were highly significant, evident from Fisher's *F*-test with high *F* value and low *P* value. Lack-of-Fit is the variation due to the model inadequacy. The lack of fit was not significant for both models (Table 3). Therefore, there is no evidence to indicate that the models do not adequately explain the variation in the responses. 

The coefficient of determination (*R* ^2^) is defined as ratio of sum of squares due to regression to the total sum of squares and is interpreted as the proportion of the variability in the data explained by the ANOVA. The values of *R* ^2^ were 0.972 and 0.954 for methanolic extraction and ethanolic extraction, respectively (Table 2), which relatively high values which imply that more than 95% of experimental data can be explained by the model. The adjusted *R* ^2^ value corrects the *R* ^2^ value for the sample size and for the number of terms in the model [21]. The values of adjusted *R* ^2^ for methanolic and ethanolic extraction were 0.936 and 0.895, respectively, which are also high and indicate a high correlation between the observed and the predicted values.  Table 4 presents a Box-Behnken design with 17 experiments as well as the experimental (*Y*_exp⁡_) and predicted response functions (*Y*_pre_) for both methanolic and ethanolic extractions. 

**Figure 1:**
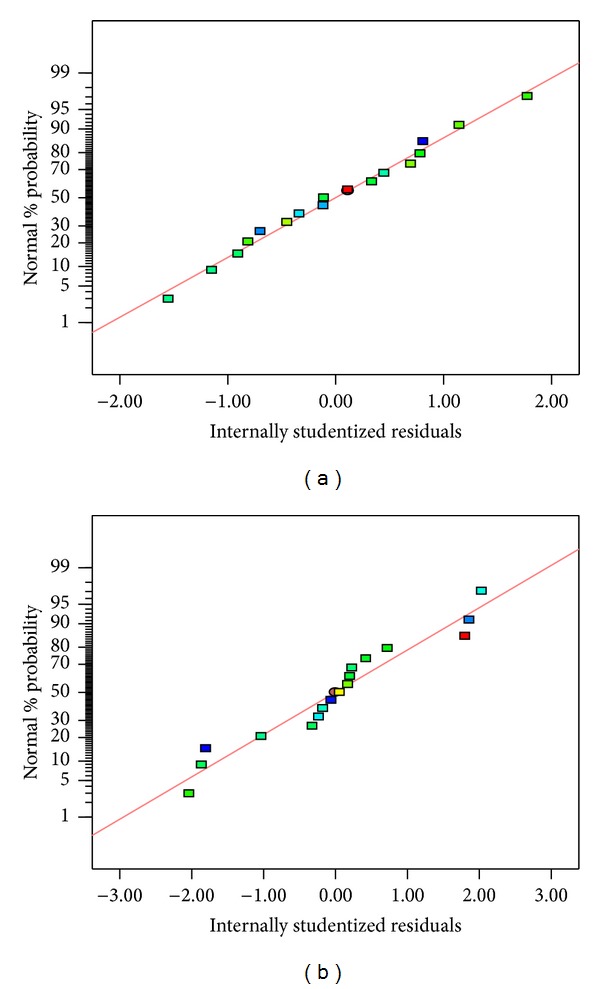
Normal probability plots of residuals for (a) methanolic extraction and (b) ethanolic extraction.

As both models showed a satisfactory fit, normal probability plot of the residuals were generated to check the normality of the residuals (Figure 1). Studentized residual is the residual divided by an estimate of its standard deviation. The residuals were studentized and values which were greater than +2 and less than −2 were considered as large. Obtaining a smaller residual value is preferred as this shows the degree of deviancy from predicted model. It is clear from Figure 1 that the residuals followed normal distribution well as majority of the data points followed the fitted line fairly closely with no reasonable outliers. 

### 3.3. Effect of Extraction Temperature, Time, and Solid to Liquid Ratio on Anthocyanin Yield

The significance of each coefficient was determined by Fisher's *F*-test and *P* value, and the larger the magnitude of *F*-value and the smaller the *P*-value, the more significant are the corresponding coefficient is. Data in Table 3 showed that, for methanolic extraction, all linear components in the experimental model were significant (*P* < 0.05) with temperature having the strongest effect on anthocyanin yield followed by solid to liquid ratio and extraction time. Positive coefficient indicated a linear effect to increase *Y* whereas negative coefficient indicated a linear effect to decrease *Y*. 

**Figure 2:**
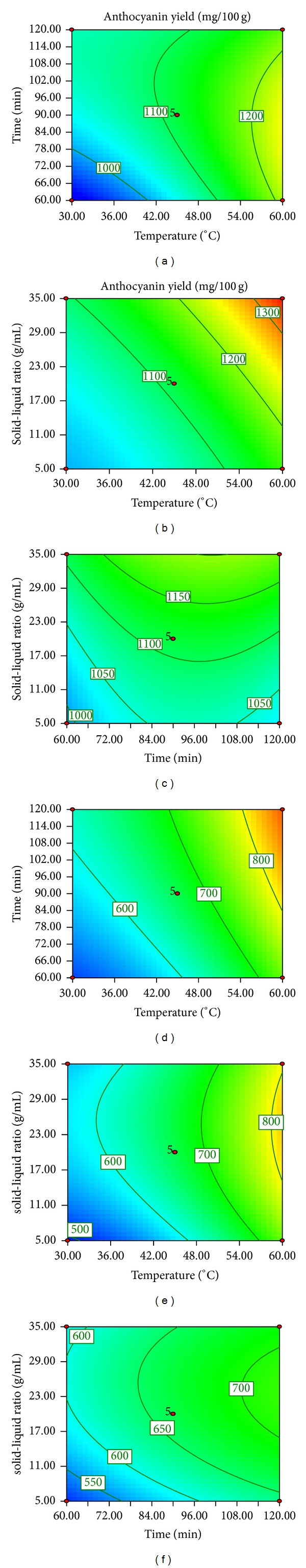
Contour plot showing the effects of variables for (a–c) methanolic extraction and (d-f) ethanolic extraction.

For methanolic extraction, the anthocyanin yield can be increased with the increase of extraction temperature as shown in Figure 2(a). The positive linear effect (*P* < 0.01) and significant negative quadratic effect (*P* < 0.05) of time have resulted in a curvilinear increase in anthocyanin yield for all the extraction time. It could be seen in Figures 2(a) and 2(c) that an increase of time beyond a certain limit of the tested range has resulted in the increase of the anthocyanin yield. As temperature and solid to liquid ratio have a stronger effect compared to time for methanolic extraction, excessive extraction time might not be effective for the extraction process. Long exposure at high temperatures would be detrimental to the desired compound as it would undergo the process of oxidation and polymerization. In addition, from an industrial point of view, longer extraction time means lower efficiency of equipment utilization [2].  Figure 2(b) showed that the increase of anthocyanin yield caused by temperature and solid to liquid ratio was almost linear. Therefore, at a fixed extraction time and by increasing the extraction temperature the result was as good as increasing the solid to liquid ratio. 

Table 3 showed that, for ethanolic extraction, the extraction temperature and time play an important role in determining anthocyanin yield. The effects of extraction temperature and time on anthocyanin yield are shown in Figure 2(d). Because both of the variables played more prominent role in the extraction efficiency for ethanolic extraction, by increasing temperature and time, the total anthocyanin content increases significantly. By heating, it gives energy to the molecules in the system to vibrate thus weakening the bond between compounds, disrupting cell membrane, and causing the compound in the cell compartment to spill out into the solvent. Figures 2(e) and 2(f) showed that the increasing of time and temperature increase anthocyanin yield with the increasing of the solid to liquid ratio up to a certain limit of the tested range. However, further increases of solid to liquid ratio did not translate into significant improvement in the yield. This can be seen in Table 3; the values of positive linear effect and negative quadratic effect of solid to liquid ratio are significant (*P* < 0.05). This observation can be explained by the fact that the system has become saturated as the solute has entirely dissolved in the fluid.

### 3.4. Perturbation Plot

**Figure 3:**
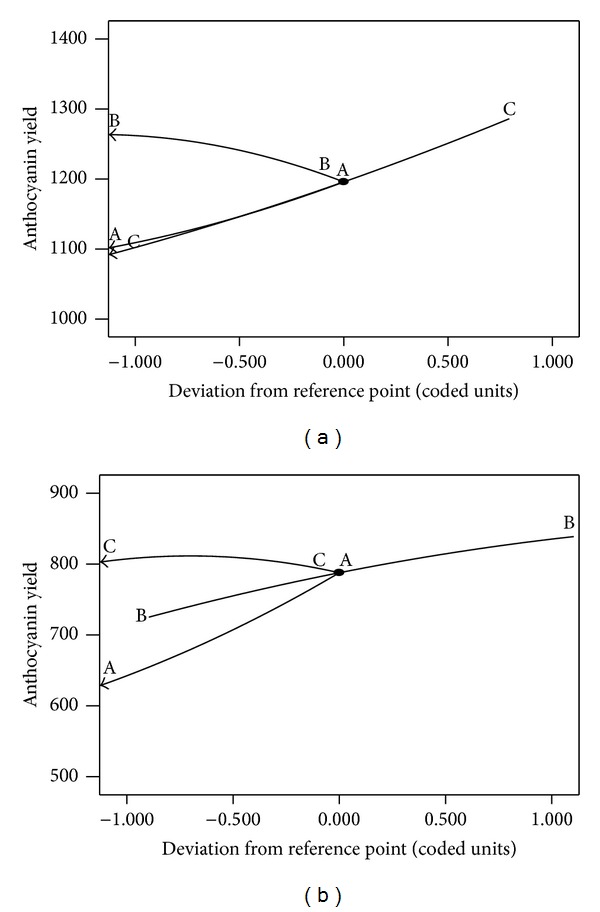
Perturbation plots for (a) methanolic extraction at temperature 60°C; time 120 min; solid to liquid ratio 23.1 g/mL and (b) ethanolic extraction at temperature 60°C; time 86.89 min; solid to liquid ratio 35 g/mL.

Pertubation plot shows how a function of a certain factor responded as the level of that factor changes, when the other factors are fixed at their optimum levels [22]. A steep slope or curvature in the plots indicates the sensitivity of the response factor [23]. Pertubation plot for methanolic and ethanolic (Figures 3(a) and 3(b), resp.) extractions were used to assess the effect of each factor on the yield. For the methanolic extraction, identical anthocyanin yield increment was observed as the temperature and solid to liquid ratio factors were increased while the yield decreases as the extraction time factor was increased. This shows that, for methanolic extraction, factors such temperature and solid to liquid ratio would influence the amount of anthocyanin extracted. For ethanolic extraction, by comparing the slope of every factor, it was seen that temperature is dominant compared to time in terms of the influence it had on anthocyanin yield. For solid to liquid ratio, it plays a minimal influence on anthocyanin yield.

### 3.5. Verification of Optimized Condition and Predictive Model

**Figure 4:**
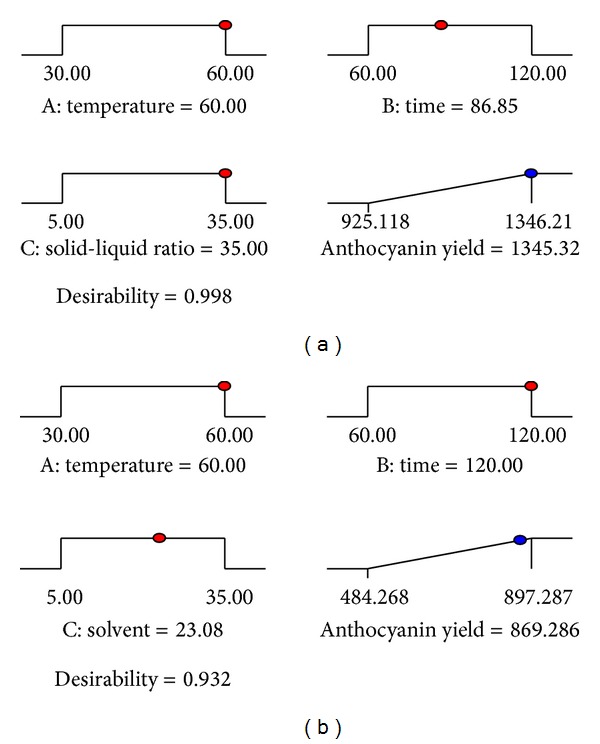
Desirability ramp of optimization for (a) methanolic extraction and (b) ethanolic extraction.

Optimization of anthocyanin extraction from *M. malabathricum* fruit was performed by using numerical optimization. The Design-Expert software used searches for a combination of factor levels that simultaneously satisfy the requirements placed on each of the responses and factors. Optimization requires that goals (i.e., none, maximum, minimum, target, or in range) are set for the variables and response where all goals then get combined into one desirability function. To find a good set of conditions that will meet all the goals, the three variables (i) extraction temperature (30°C–60°C), (ii) extraction time (60–120 min), and (iii) solid to liquid ratio (g/mL) were set within range while anthocyanin yield was set at maximum. The “importance” of goals (option 1–5) for all variables was considered to be equally important in a setting of 3. For response, the “importance” was set at 5 in order to meet the objective of getting maximum anthocyanin yield. By applying the desirability function approach, the optimum level of various parameters was obtained as showed in Table 5.  Figure 4 showed desirability ramps that were developed from optimum points via numerical optimization.

A triplicate experiment was set up to validate the optimized condition. As shown in Table 5, the experimental data were in good agreement with the predicted values for methanolic and ethanolic extractions. Relative error between predicted and experimental values fell at 0.066% (1346.208 mg/100 g) and 1.321% (880.923 mg/100 g) for methanolic and ethanolic extractions, respectively. The verification value for anthocyanin yield obtained is within 99% of predicted values which clearly showed that the model fitted the experimental data very well and therefore optimized the anthocyanin extraction efficiently within the specified range of process parameters.

### 3.6. Identification of Anthocyanin

**Figure 5:**
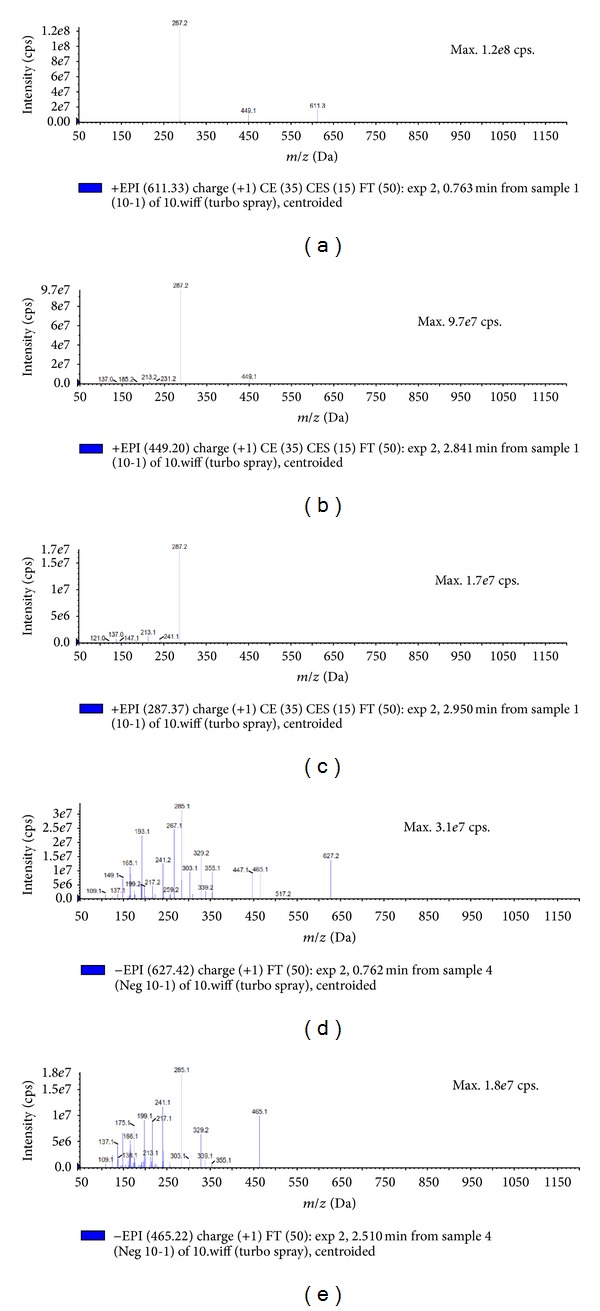
Mass spectra of anthocyanin-rich extract after gel filtration using Sephadex LH-20: (a–c) positive mode and (d-e) negative mode.

Figure 5 showed the mass spectra for (Figures 5(a)–5(c)) positive mode and (Figures 5(d)-5(e)) negative mode of anthocyanin-rich extract of *M. malabathricum* by UPLC-ESI-MS/MS.  Figure 5(a) (retention time *t*_*R*_ = 0.763 min), with *M* ^+^ at *m/z* 611.3, was identified as cyanidin dihexoside and fragments ions at *m/z* 449.1 and *m/z* 287.2 corresponded to cyanidin.  Figure 5(b) (*t*_*R*_ = 2.841 min), with *M* ^+^ at *m/z* 449.1, was identified as cyanidin hexoside and fragment ions at *m/z* 287.2.  Figure 5(c) (*t*_*R*_ = 2.950 min), with *M* ^+^ at *m/z* 287.2, was identified as cyanidin aglycone.  Figure 5(d) (*t*_*R*_ = 0.762 min), with *M* ^−^ at *m/z* 627.2, was identified as delphinidin dihexoside and fragments ions at *m/z* 465.1 and *m/z* 303.1 corresponded to delphinidin.  Figure 5(e) (*t*_*R*_ = 2.510 min), with *M* ^−^ at *m/z* 465.1, was identified as delphinidin hexoside and fragment ions at *m/z* 303.1.

## 4. Conclusion

The experimental design approach using RSM was successfully applied in the optimization of anthocyanins from *M. malabathricum* fruit. Under optimum condition, methanolic extraction showed the highest anthocyanin yield which was 1345.32 mg/100 g compared to ethanolic extraction, 869.29 mg/100 g. By using the optimum condition established, anthocyanin pigments from methanolic extraction can be applied as natural colourants in coating products, textiles, and solar cell industry whereas anthocyanin pigments from ethanolic extraction are recommended for food applications due to thier GRAS (generally recognized as safe) qualification. In this study, among three parameters tested, temperature was found to be the most prominent factor affecting the efficiency of anthocyanin extraction. *M. malabathricum* fruit was tentatively identified to contain cyanidin dihexoside, cyanidin hexoside, cyanidin, delphinidin dihexoside, and delphinidin hexoside.

## Figures and Tables

**Table 1:** Independent variables and their levels used for Box-Behnken design.

Variables	Factors	Levels		
	*X*	−1	0	1
Extraction temperature, (°C)	*X*_1_	30	45	60
Extraction time, (min)	*X*_2_	60	90	120
Solid to liquid ratio, (g/mL)	*X*_3_	0.5 : 5	0.5 : 20	0.5 : 35

**Table 2:** Adequecy of model tested.

Source	Sum of squares	df	Mean square	*F* Value	Prob > *F*	Remarks
Sequential model sum of squares for acidified methanolic extraction						
Mean versus Total	20893255.04	1	20893255.04			
Linear versus Mean	128877.78	3	42959.26	20.25	<0.0001	
2FI versus Linear	10748.42	3	3582.81	2.13	0.1600	
Quadratic versus 2FI	12433.06	3	4144.35	6.60	0.0189	Suggested
Cubic versus Quadratic	874.80	3	291.60	0.33	0.8045	Aliased
Residual	3520.73	4	880.18			
Total	**21049709.83**	**17**	**1238218.23**			

Sequential model sum of squares for acidified ethanolic extraction						
Mean versus Total	7196887.80	1	7196887.80			
Linear versus Mean	147605.80	3	49201.93	28.80	<0.0001	Suggested
2FI versus Linear	532.96	3	177.65	0.08	0.9683	
Quadratic versus 2FI	13908.91	3	4636.30	4.18	0.0544	Suggested
Cubic versus Quadratic	6069.16	3	2023.05	4.76	0.0829	Aliased
Residual	1699.43	4	424.86			
Total	**7366704.07**	**17**	**433335.53**			

Source	Std. Dev.	*R* ^2^	Adjusted *R* ^2^	Predicted *R* ^2^	PRESS	Remarks

Model summary statistics for acidified methanolic extraction						
Linear	46.058	0.824	0.783	0.654	54157.757	
2FI	41.023	0.892	0.828	0.542	71629.308	
Quadratic	25.059	0.972	0.936	0.875	19498.004	Suggested
Cubic	29.668	0.977	0.910		+	Aliased

Model summary statistics for acidified ethanolic extraction						
Linear	41.334	0.869	0.839	0.741	43972.027	Suggested
2FI	46.559	0.872	0.796	0.406	100897.766	
Quadratic	33.314	0.954	0.895	0.413	99761.992	Suggested
Cubic	20.612	0.990	0.960		+	Aliased

+ Case(s) with leverage of 1.0000: PRESS statistic not defined.

**Table 3:** ANOVA for response surface quadratic model.

Source	Coefficient estimate	Sum of squares	df	Mean square	*F*-value	*P*-value	Remarks
Acidified methanolic extraction							
Model	1114.595	152059.256	9	16895.473	26.906	0.0001	Significant
*X*_1_	103.359	85464.893	1	85464.893	136.105	<0.0001	
*X*_2_	26.475	5607.274	1	5607.274	8.930	0.0203	
*X*_3_	68.744	37805.617	1	37805.617	60.206	0.0001	
*X*_12_	−45.922	8435.296	1	8435.296	13.433	0.0080	
*X*_13_	22.196	1970.579	1	1970.579	3.138	0.1198	
*X*_23_	9.254	342.543	1	342.543	0.546	0.4842	
*X*_1_^2^	24.728	2574.683	1	2574.683	4.100	0.0825	
*X*_2_^2^	−48.608	9948.234	1	9948.234	15.843	0.0053	
*X*_3_^2^	11.160	524.442	1	524.442	0.835	0.3912	
Residual		4395.537	7	627.934			
Lack of fit		874.804	3	291.601	0.331	0.8045	Not significant
Pure error		3520.733	4	880.183			
Cor total		156454.793	16				

Acidified ethanolic extraction							
Model	664.170	162047.676	9	18005.297	16.224	0.0007	Significant
*X*_1_	118.806	112918.450	1	112918.450	101.747	<0.0001	
*X*_2_	57.681	26616.436	1	26616.436	23.983	0.0018	
*X*_3_	31.763	8070.915	1	8070.915	7.272	0.0308	
*X*_12_	7.237	209.468	1	209.468	0.189	0.6770	
*X*_13_	−3.966	62.917	1	62.917	0.057	0.8186	
*X*_23_	−8.071	260.580	1	260.580	0.235	0.6428	
*X*_1_^2^	31.220	4103.958	1	4103.958	3.698	0.0959	
*X*_2_^2^	−11.849	591.175	1	591.175	0.533	0.4892	
*X*_3_^2^	−48.100	9741.404	1	9741.404	8.778	0.0210	
Residual		7768.593	7	1109.799			
Lack of fit		6069.165	3	2023.055	4.762	0.0829	Not significant
Pure error		1699.428	4	424.857			
Cor total		169816.269	16				

**Table 4:** Box-Behnken design arrangement and responses.

Run	*X*_1_	X_2_	X_3_	Anthocyanin yield* (mg/100 g)			Anthocyanin yield* (mg/100 g)		
				(acidified methanolic extraction)			(acidified ethanolic extraction)		
				*Y*_exp⁡_	*Y*_pre_	% sd	Y_exp⁡_	*Y*_pre_	% sd
1	45	90	20	1132.18	1114.60	3.40	654.60	664.17	2.65
2	60	120	20	1164.47	1110.16	1.32	897.29	867.26	3.46
3	30	60	20	925.12	1114.60	1.10	484.27	514.29	8.36
4	45	90	20	1154.45	1114.60	2.84	633.44	664.17	1.85
5	45	60	5	982.45	1114.60	2.75	537.70	506.71	2.19
6	45	90	20	1094.33	1000.58	1.74	685.77	664.17	3.13
7	30	120	20	1065.39	1213.52	4.05	612.29	615.18	4.41
8	45	120	5	1021.42	1025.63	3.10	642.07	638.21	2.08
9	45	90	20	1079.86	1162.90	4.65	676.86	664.17	4.25
10	60	90	5	1177.27	914.96	2.87	704.42	738.30	3.95
11	45	120	35	1190.35	1181.62	4.29	654.60	685.59	1.79
12	45	60	35	1114.37	991.18	4.28	582.51	586.38	7.79
13	30	90	5	999.15	1344.78	2.65	491.78	492.76	1.18
14	60	60	20	1207.89	1114.60	3.10	740.32	737.43	9.90
15	30	90	35	1079.31	1093.67	7.93	598.10	564.21	8.31
16	60	90	35	1346.21	1059.75	5.70	794.87	793.89	0.00
17	45	90	20	1112.15	1174.63	3.16	670.18	664.17	3.32

*Data are presented as mean of triplicate analyses.

*X*_1_: temperature; *X*_2_: time; *X*_3_: solid-liquid ratio.

% sd < 10 is considered significant.

**Table 5:** Experiment confirmation of predicted value at optimal extraction condition.

Optimal levels	Anthocyanin yield (mg/100 g)					
	*Y*_predicted_	Experimental value			Mean*	Relative error^a^ (%)
		1	2	3		
Acidified methanolic extraction	1345.320	1297.503	1437.774	1303.348	1346.208	0.066
*X*_1_ = 60°C						
*X*_2_ = 120 min						
*X*_3_ = 0.5 g : 23.1 mL						
Acidified ethanolic extraction	869.290	878.355	886.060	878.355	880.923	1.321
*X*_1_ = 60°C						
*X*_2_ = 86.89 min						
*X*_3_ = 0.5 g : 35 mL						

^a^Relative error (%) = [(experimental value − predicted value)/experimental value] × 100%.

Mean is average value from triplicate of experimental run.

## Abbreviations

RSM: Response surface methodology
UPLC-ESI-MS/MS: Ultra performance liquid chromatography electrospray tandem mass spectrometry
BBD: Box-Behnken design.
